# Neuronal Cell-type Engineering by Transcriptional Activation

**DOI:** 10.3389/fgeed.2021.715697

**Published:** 2021-09-01

**Authors:** Songlei Liu, Johannes Striebel, Giovanni Pasquini, Alex H. M. Ng, Parastoo Khoshakhlagh, George M. Church, Volker Busskamp

**Affiliations:** ^1^ Department of Genetics, Blavatnik Institute, Harvard Medical School, Boston, MA, United States; ^2^ Wyss Institute for Biologically Inspired Engineering, Harvard University, Boston, MA, United States; ^3^ Department of Ophthalmology, Medical Faculty, University of Bonn, Bonn, Germany; ^4^ GC Therapeutics, Inc, Cambridge, MA, United States

**Keywords:** ORF, neuron, forward programming, transcription factor, CRISPRa

## Abstract

Gene activation with the CRISPR-Cas system has great implications in studying gene function, controlling cellular behavior, and modulating disease progression. In this review, we survey recent studies on targeted gene activation and multiplexed screening for inducing neuronal differentiation using CRISPR-Cas transcriptional activation (CRISPRa) and open reading frame (ORF) expression. Critical technical parameters of CRISPRa and ORF-based strategies for neuronal programming are presented and discussed. In addition, recent progress on *in vivo* applications of CRISPRa to the nervous system are highlighted. Overall, CRISPRa represents a valuable addition to the experimental toolbox for neuronal cell-type programming.

## Introduction to CRISPR-Cas Transcriptional Activation

Induced expression of desired genes has been an important strategy for revealing gene function and for modulating cellular activity for synthetic biology and therapeutic applications. Apart from ectopically expressing additional copies of a gene by introducing their open reading frames (ORFs), methods to artificially activate endogenous copies of genes have been explored, including transcription activating factors tethered to zinc finger proteins (Beerli et al., 2000) and transcription activator-like effectors (TALE) (Miller et al., 2011; Zhang et al., 2011; Maeder et al., 2013b; Perez-Pinera et al., 2013b). Originally discovered as a virus-resistance mechanism from bacteria (Barrangou et al., 2007), the CRISPR-Cas system has provided efficient, precise, and scalable ways to modulate expression of genes, and has been successfully adopted for targeted gene activation (Mali et al., 2013; Perez-Pinera et al., 2013a; Maeder et al., 2013a; Cheng et al., 2013; Tanenbaum et al., 2014; Zalatan et al., 2015; Konermann et al., 2015; Chavez et al., 2015, 2016).

**FIGURE 1:**
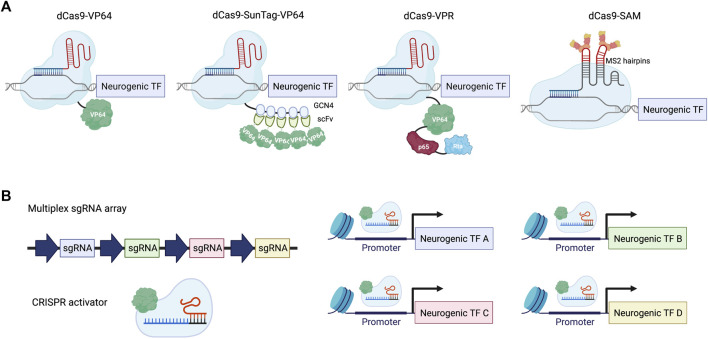
Neuronal differentiation using CRISPRa. **(A)** Examples of the CRISPRa system. dCas9-VP64: dCas9 tagged with a single copy of VP64 activation domain; dCas9-SunTag-VP64: dCas9 tagged with a tandem repeat of GCN4 peptide, which recruits multiple copies of VP64 through scFv binding; dCas9-VPR: dCas9 fused with a combination of activator domains: VP64, p65 and Rta; dCas9-SAM: gRNA is linked with two copies of the MS2 coat protein binding-RNA stem loop to recruit MS2-VP64 for gene activation. **(B)** Scheme illustrating multiplexed activation of neurogenic transcription factors (TFs) using CRISPRa.

To achieve gene activation with CRISPR-Cas9, a catalytically deactivated Cas9 (dCas9) was created to bind to specific genomic regions without the ability to create a double-strand break (Jinek et al., 2012; Gasiunas et al., 2012; Qi et al., 2013; Konermann et al., 2013; Mali et al., 2013). To endow dCas9 with the power to induce gene expression, different transcriptional activator domains have been explored for their strength of gene activation (Figure 1A). The first generation of CRISPRa was inspired by zinc finger and TALE-based approaches and used single activator domains, including VP64 or p65. VP64 consists of four copies of VP16, which is a transcriptional activator derived from the herpes simplex virus. p65 is part of the NF-κB complex and is responsible for transcriptional activation in immune signaling. The second generation of CRISPRa systems devised different strategies to recruit multiple copies of different activators, including a SunTag array for recruiting 10 or 24 copies of VP64 to a given locus, a tandem fusion of VP64, p65, and Rta (VPR) to dCas9, and an MS2 hairpin/coat protein interaction for additional copies of activators. By introducing multiple single guide RNAs (sgRNAs) into the same cell, multiplexed gene activation can be achieved using CRISPRa (Figure 1B). For a more comprehensive discussion on the development of CRISPRa, we would like to point readers to reviews previously published on this topic (La Russa and Qi, 2015; Thakore et al., 2016; Xu and Qi, 2019; Pandelakis et al., 2020; Shakirova et al., 2020). In this review, we summarize recent *in vitro* and *in vivo* applications of CRISPRa with a particular emphasis on neuronal cell fate engineering in comparison to ORF expression strategies.

## Targeted Transcription Factor Activation for Neuronal Differentiation

The *in vitro* engineering of cell types that are present in the human body has many important applications, including studying cellular functions, investigation of disease progression, development of therapeutic interventions, and cell-based transplantation therapies. Being recognized for their key roles in modulating gene expression, TFs have been broadly used for cell fate engineering, most of which utilized cDNA or ORF overexpression. Pioneering work in 1987 showed that cell identity could be altered by forced expression of a single TF MyoD, which converted fibroblasts into myoblasts (Davis et al., 1987). Just over a decade ago, the overexpression of four TFs (Oct3/4, Sox2, c-Myc, and Klf4) was sufficient to reprogram fibroblasts into induced pluripotent stem cells (iPSCs) (Takahashi and Yamanaka, 2006). Shortly after that, three TFs (Ascl1, Brn2, Myt1l) were demonstrated to convert cells between lineages, from fibroblasts to neurons (Vierbuchen et al., 2010). Since then, the forced expression of TFs has been shown to produce more specific types of neurons, including motor neurons (Son et al., 2011), dopaminergic neurons (Pfisterer et al., 2011), sensory neurons (Wainger et al., 2015), and striatal neurons (Victor et al., 2014). More recently, the TFome, a comprehensive human TF ORF library, was constructed and screened for its ability to induce human iPSC (hiPSC) differentiation, which revealed additional routes to produce neurons (Ng et al., 2021). For a more extensive discussion on neuronal programming with TFs, we point readers to specific reviews (Masserdotti et al., 2016; Carter et al., 2020; Flitsch et al., 2020). We would also like to point out that TFs are not the only target of interest in neuronal programming, it has been shown that introducing genes regulating cellular metabolism can also facilitate the process of neuronal conversion (Yanes et al., 2010; Gascón et al., 2016; Quadrato et al., 2016; Russo et al., 2021).

The aforementioned studies used ORF overexpression of exogenous copies of TFs, but with the CRISPR-Cas system, it is possible to activate endogenous genes, which may have advantages over ORF overexpression. CRISPRa-based cell programming has been demonstrated successfully in a number of studies. By targeting NEUROG2 or NEUROD1 using dCas9-VPR with a lentiviral pool of 30 gRNAs, hiPSCs were rapidly differentiated into cells with neuronal morphology after 4 days post induction (dpi) (Chavez et al., 2015). These differentiated cells were also positive for neuronal markers including beta III tubulin (TUBB3) and neurofilament 200 (NEFH). Of note, the second generation dCas9-VPR was needed, as VP64 fused to dCas9 targeting NEUROG2 or NEUROD1 activation was not successful for neuronal differentiation. Previous studies have demonstrated that ectopic expression of Neurog2 or NeuroD1 ORFs were sufficient to induce nearly complete neuronal differentiation (Zhang et al., 2013). This highlights the importance of achieving strong gene activation in the context of cell fate engineering. Indeed, it was shown by qRT-PCR experiments that dCas9-VPR induced 10 times higher NEUROG2 and 18 times higher NEUROD1 mRNA expression than dCas9-VP64, suggesting stronger gene activation led to more robust cell differentiation.

One potential advantage of the CRISPRa system relates to epigenetic modifications around the endogenous loci, which are hypothesized to better mimic natural epigenetic marks. Evidence for this remodeling process was investigated by delivering gRNAs targeting promoter regions of Ascl1, Brn2 and Myt1l to convert mouse embryonic fibroblasts (MEFs) into neurons (Black et al., 2016). A dCas9 with VP64 domains fused on both N-terminal and C-terminal (VP64-dCas9-VP64) was used in this study. Neuronal differentiation resulting from the CRISPRa system and direct TF ORF expression was compared. When the plasmids were introduced transiently, the CRISPRa system showed a stronger induction of the endogenous loci, while total mRNA expression, measured by qRT-PCR with primers not distinguishing endogenous or exogenous transcripts, was higher using ORFs. Also, under transient transfection conditions, a higher level of chromatin remodeling including stronger ChIP-qPCR signals of H3K27ac and H3K4me3 at endogenous Ascl1, Brn2 and Myt1l loci was observed by using the CRISPRa system expression compared to ORF expression. However, when CRISPRa or ORFs were stably integrated into the genome using lentiviral vectors, ORF expression also promoted chromatin remodeling at endogenous loci, indicating potential feedback regulation of the endogenous loci. Even though gRNAs still induced higher transcription from endogenous Brn2 and Ascl1 loci (while gRNAs for Myt1l were less robust), constitutive expression of the three ORFs produced a significantly higher percentage of Tubb3+/Map2+ cells. These results indicate that in order to achieve a similar level of differentiation using CRISPRa when compared with ORF expression, design of more robust gRNAs and stronger dCas9-fused activators are necessary.

## Multiplexed TF Screening for Neuronal Programming

**FIGURE 2:**
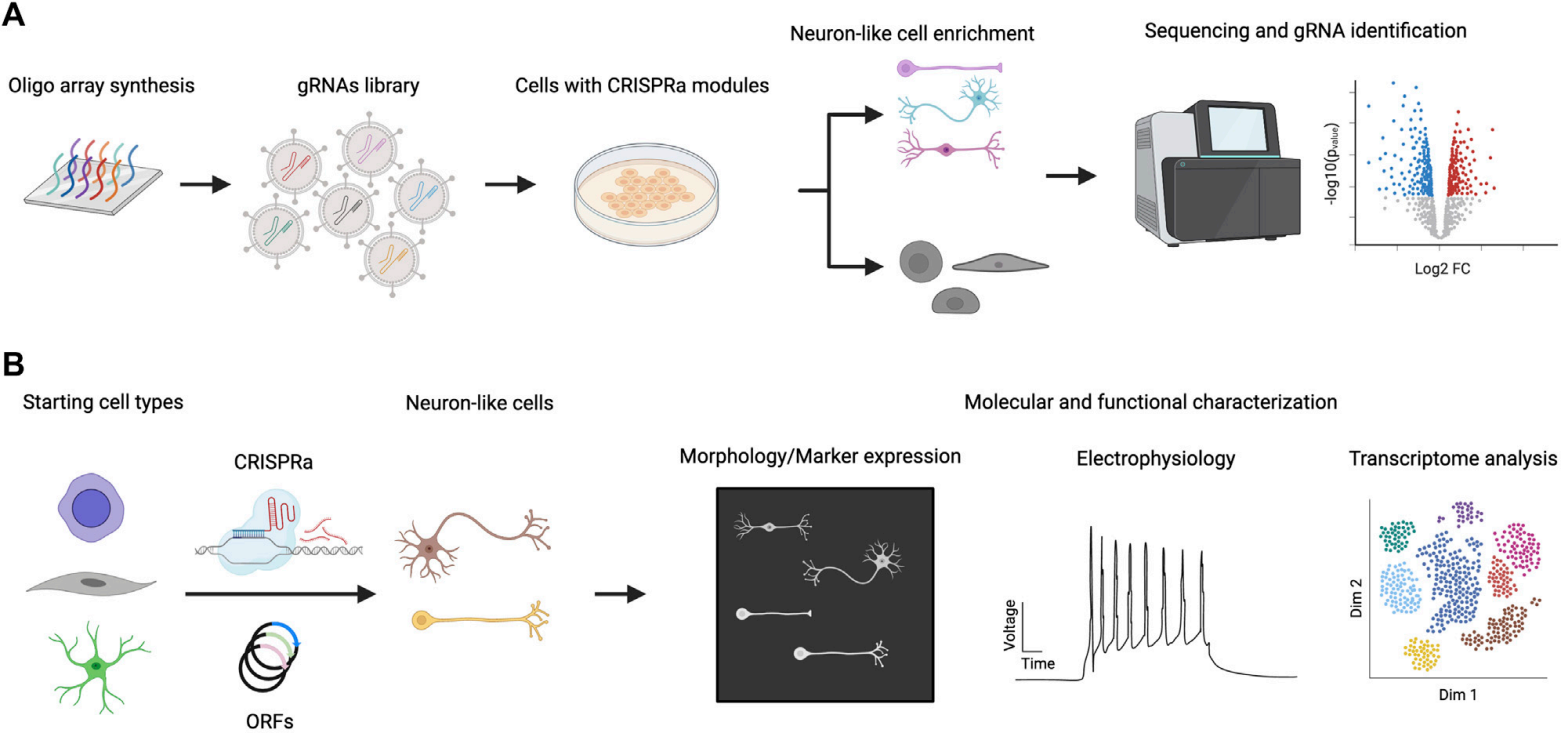
CRISPRa screen for neuronal differentiation and downstream characterization. **(A)** A library of gRNAs is synthesized (e.g. chip oligo array synthesis) and delivered to cells expressing CRISPRa proteins. Cells that exhibit neuronal characteristics are enriched and their gRNA distribution is compared with non-neuronal populations. gRNAs that are specifically enriched in neuronal populations are considered hits of the screen. **(B)** Hits from CRISPRa screen are validated by focused study using gRNAs targeting or ORFs expressing specific genes. Further characterization of the induced neuronal cells includes morphological, electrophysiological and transcriptomic studies.

The CRISPR-Cas system is easily scalable to highly multiplexed screens using complex gRNA libraries because of the short length of gRNAs compared to full-length TF ORFs (Figure 2). In this section, we will discuss recent examples of multiplexed TF screenings in the context of neuronal differentiation (Table 1). A CRISPRa screen in mouse embryonic stem cells (ESCs) was performed in which 2,428 computationally predicted TFs were targeted with 55,561 gRNAs (Liu et al., 2018). The CRISPRa strategy used in this study was dCas9-fused with SunTag plus scFv-fused VP64. The gRNA library was introduced by lentiviral transduction. To enrich for neuronal-promoting genes, endogenous Tubb3 was tagged with human CD8, which was later used as a marker for cell sorting, followed by next-generation sequencing (NGS) analysis of enriched gRNAs through targeted amplification of gRNA integration loci and sequencing. To rank gRNAs on their neuronal promoting strength, the authors developed an algorithm that calculates the probabilistic contribution of each factor to neuronal differentiation. From this single-factor screen, 74 genes that positively contributed to neuronal differentiation were discovered. When the transcriptome of primary neurons and ESCs were compared, 41 out of these 74 genes exhibited no differential expression (DE) between neurons and ESCs. As DE is one strategy to select TF candidates for screening, this points to the limitation of relying solely on this strategy for the selection of candidate TFs for neuronal induction. Top hits of the screen were validated by overexpressing ORFs encoding those TFs to rule out the possibility of off-target effects. This also provides an opportunity to compare the strength of differentiation between CRISPRa and ORFs. Based on the percentage of PSA-NCAM + cells after 12 days, lentiviral-introduced gRNA and ORFs generated a comparable percentage of PSA-NCAM + cells upon induction; however, further systematic studies will be needed to comprehensively generalize this comparison. Features such as the expression level of ORFs (which can induce supraphysiological expression higher than CRISPRa), the species tested (as human cells appear more difficult to program than murine cells), and the cell type generated (beyond neurons) should be investigated. In addition to single-factor screening, a pairwise TF screen for the top 19 factors was performed to study the genetic interaction between these 171 pairs for gRNAs, and a strong synergistic effect between Ezh2 and Brn2 was observed. Ezh2+Brn2 converted fibroblasts to neurons, while neither alone had that effect. Notably, the direct conversion was only demonstrated for ORF expression but not for CRISPRa.

**TABLE 1:** Examples of multiplexed TF screening for neuronal programming.

Study	Type	Target number	Screening system	Screening hits
Liu et al. (2018)	CRISPRa	55,561 gRNAs targeting 2,428 mouse TFs, pooled	Mouse ESCs, lentivirus, 12-days Doxycycline (Dox) induction	74 TFs
Tsunemoto et al. (2018)	cDNA	59 TFs (57 mouse and 2 human) tested as 598 pairs of TFs, arrayed	Mouse embryonic fibroblasts (MEFs), lentivirus, 2 weeks (8-days Dox from Day 0)	76 TF pairs
Parekh et al. (2018)	ORF	61 TFs, pooled; 14-element neural TF sub-library	Human ESCs, lentivirus, 5–6 days expression	Unbiased single-cell analysis
Black et al. (2020)	CRISPRa	8,435 gRNAs targeting 1,496 TFs, pooled	Human iPSCs, lentivirus, 5-days Dox induction	17 TFs
Ng et al. (2021)	ORF	1,732 human TF splice isoforms, pooled	Human iPSCs, lentivirus, 4-days Dox induction	290 TFs. 11 neuronal TFs; Pairs and combinatorial creens ongoing

CRISPRa screening for neuronal differentiation has also been carried out in hiPSCs by using a library of 8,435 gRNAs targeting 1,496 TFs in the human genome. A dCas9 with VP64 fused to both N and C termini was used for gene activation and a TUBB3-2A-mCherry reporter was generated for indicating neuronal cell fate (Black et al., 2020). After comparing gRNA distributions in mCherry-high- and mCherry-low-expressing cells, 17 TFs were identified as neurogenic factors by having at least two gRNAs enriched in mCherry-high cells, including the known neurogenic TFs NEUROD1, NEUROG1, and NEUROG2. Of note, another well-characterized neurogenic TF, ASCL1, was not selected because only one out of five gRNAs was enriched. Again, this points to the necessity of gRNA design optimization and usage of redundant gRNAs in a CRISPRa screen. After the single-factor screen, a focused paired-TF screen by combining NEUROG3 or ASCL1 with each of the remaining TFs in the gRNA library was performed in order to identify factors that could promote or inhibit neuronal differentiation. The majority of positive regulators for NEUROG3 or ASCL1-mediated neuronal differentiation overlapped, including genes that were not observed in the single-factor screen: LHX6, LHX8, and HMX2. However, synergistic effects specific to NEUROG3 or ASCL1 were also identified, exemplified by FEV, a TF expressed in serotonergic neurons (Kriegebaum et al., 2010), only showed synergy in the NEUROG3 group, whereas NKX2.2, a homeobox protein involved in brain and pancreas development, only enriched with ASCL1. Downstream characterizations of the gRNA hits for neuronal differentiation, including immunofluorescence, RNA-sequencing, electrophysiological analysis, were performed by overexpressing ORFs encoding the TFs, illustrating the complementarity of the two approaches.

Parallel to the development of CRISPRa systems, overexpression of ORFs has also been used for gain-of-function screens for neuronal differentiation. Five hundred and ninety eight TF pairs were tested for their ability to differentiate MEFs to induced neurons (iNs) (Tsunemoto et al., 2018). TF pairs were screened in an arrayed format using doxycycline-inducible lentiviral vectors. From the screen, 76 out of 598 TF pairs could convert MEFs to neurons, which showed neuronal morphologies, expressed neuronal markers and possessed electrical activity. Neurog3/Pou5f1- and Neurod2/Pou4f3-induced neurons exhibited excitatory postsynaptic currents after 16–24 dpi in the absence of glia co-culture. By performing bulk transcriptome analysis of iNs and comparing with MEFs and *in vivo* neurons, a 75.5% overlap of enriched genes was observed when comparing iNs or *in vivo* neurons to MEFs. However, it was unclear whether the magnitude of enrichment (i.e. expression fold-change and/or significance) was similar, but these results do highlight the convergence to a “core neuronal transcriptome” when diverse TF pairs are used. Because endogenous neurons have many subtypes, the heterogeneity amongst iNs created from different TF pairs was investigated to see if different TF pairs could produce different subtypes of neurons. Groups of iNs that expressed higher levels of nicotinic receptor genes were identified, also exhibiting preferred responses to nicotine stimulation in calcium imaging experiments. Overall, only a limited number of genes provided a confident similarity of resulting iN cells to endogenous subtypes. This observation indicates that even though different pairs of TFs could produce different gene expression profiles on top of the core neuronal programs, they are not yet specific enough to produce the vast diversity of neurons that exist in the mammalian brain. Combinatorial and serial screening of TFs for recreating neuron subtypes *in vitro* is still in its early stages. Although different pairs of TFs can reprogram fibroblasts into induced neurons, it is important to note that even the same pair of factors can reprogram other non-neuronal cells into different types of neurons: a study (Karow et al., 2018) showed that Ascl1 and Sox2 can reprogram human brain pericytes into different neurons, possibly as each cell is being influenced by different signaling pathways in the milieu of other cells undergoing reprogramming. The efforts on comprehensive single-cell transcriptomic analysis of the brain will also provide guidance to the engineering process, by elucidating the diverse cellular identities in the brain and their genetic underpinnings (Hodge et al., 2019).

Combining single-cell transcriptomic readout with pooled TF activation in stem cells is a high-throughput way to establish causality between TFs and their effects on cell fate. Along this line, a proof of concept screen with 61 barcoded ORFs demonstrated the feasibility of associating TF expression with specific single-cell transcriptome clusters (Parekh et al., 2018). Clusters enriched with NEUROD1, NEUROG1 and NEUROG3 were observed, in which gene modules known for neuronal differentiation were upregulated. A more focused analysis with 14 pooled neural TFs further revealed their transcriptomic effects. Pooled screens like this, combined with the accumulating amount of primary brain single-cell transcriptome data, could provide broad and deep knowledge about how the vast diversity of neuronal identity can be recreated with targeted transcriptional activation.

As previously discussed, CRISPRa screens targeting nearly all TFs in the human or mouse genome (Liu et al., 2018; Black et al., 2020) have been enabled in principle by the ease of synthesizing large-scale gRNA libraries. However, genome-wide screening of TFs using ORFs has until recently been hindered by the lack of a complete TF ORF library. This was overcome by a comprehensive human TF ORF library (TFome) consisting of 1564 TF genes and 1732 TF splice isoforms (Ng et al., 2021). Using a doxycycline-inducible lentiviral expression system, the TFome was systematically screened for induction of differentiation in three independent hiPSC lines with a low multiplicity of infection of 0.1 to ensure that each cell expressed at most one TF. To enable lineage-agnostic screening, after 4 dpi, differentiated cells marked by lower expression of pluripotency marker TRA-1-60 were enriched and sequenced for integrated TFs. A total of 290 TFs were identified to induce differentiation in at least two hiPSC lines. Among the top hits of the screen are ATOH1, NEUROG1, and ASCL1, which have previously been reported for inducing neuronal differentiation (Busskamp et al., 2014; Chanda et al., 2014; Xue et al., 2019). During validation of the screening hits, stronger differentiation using the PiggyBac transposon system compared to lentiviral transduction was reported, which is likely due to a higher copy number of ORFs by PiggyBac integration. An average of 10–15 copies of each TF integrated per cell was observed. One advantage of using ORFs *versus* CRISPRa is the ability to elevate expression of a certain TF isoform. When the four isoforms of ETV2 were compared for inducing endothelial cell differentiation, only isoform 2 was able to generate cells that have near complete VE-cadherin expression and possess high tubulogenic capacity. ETV2-programmed cells are useful for engineering vascularized brain organoids (Cakir et al., 2019; Skylar-Scott et al., 2020). Neuronal differentiation induced by ATOH1 in a cell-autonomous manner (i.e. without changes to media conditions) was further characterized. Rapid neurogenesis was reflected by over 98% of cells having NCAM expression after 4 days, and by transcriptomic similarity observed from principal component and CellNet analysis. At 14 dpi, the cells showed trains of action potentials in response to current injection, indicating mature neuronal function. These results demonstrate the power of ORF-based approaches such as the TFome for genome-scale screens and for ORFs to serve as the gold standard used for validation and functional characterization of programmed cells.

## *In vivo* Applications of CRISPRa in the Nervous System

CRISPRa is an established tool for targeted activation of genes *in vitro*. After demonstrating the possibility of *in vivo* CRISPR-Cas gene editing in the brain, the usage of CRISPRa in the nervous system has only recently been investigated more broadly. A pioneering *in vivo* use of CRISPRa targeting the brain was reported in 2018 (Zhou et al., 2018). The study used CRISPRa for simultaneous activation of multiple genes. A system of two adeno-associated viruses (AAVs) was used for delivering gRNAs and activating dCas9 in a transgenic mouse model. Astrocytes were converted into functional neurons in the mouse midbrain by targeting Ascl1, Neurog2 and Neurod1. Furthermore, it was demonstrated that gRNA arrays are able to activate various targets at once.

Several other studies further demonstrated successful CRISPRa applications in animal models. Two studies have investigated the use of CRISPRa as a method to counteract epilepsy *via* overexpression of certain ion channels in neurons (Colasante et al., 2020; Yamagata et al., 2020). The first study has shown that the upregulation of the gene Kcna1 coding for a potassium channel decreased neuronal excitability and led to a decrease in spontaneous seizures in a mouse model of focal temporal lobe epilepsy (Colasante et al., 2020). In the second study, inhibitory neurons were targeted in a mouse model of Dravet syndrome to increase the expression of Scn1a (Yamagata et al., 2020). This led to a reduction of seizures and partial improvement of behavioral measures. In the first study, both dCas9 and the gRNA cassette were delivered using AAVs. The second study relied on transgenic mice for dCas9 expression. In a second step, gRNAs were then transduced *via* AAVs.

Generally, the use of ORFs has some advantages for *in vivo* applications compared to CRISPRa strategies as ORFs from the same species are generally used. It has been shown that Cas9 induced host responses in mice (Chew et al., 2016) and in one study, 78% of the 125 human adult blood donors tested had antibodies against Cas9 proteins (Charlesworth et al., 2019). Regarding CRISPRa, the dCas9 system would eliminate the risk for severe off-target effects because no cleavage of DNA occurs. Nevertheless, there is the possibility of off-target interference with gene expression. Lastly, relying on the endogenous loci *in vivo* could be hampered by any mutations or single nucleotide polymorphisms in those genes unless they were identified and corrected prior to gene activation using CRISPRa.

Delivery of CRISPR components or ORFs into targeted neurons is limited by the specific method used. Classically they are delivered using viral vectors such as lentiviruses or AAVs, but other non-viral methods are also evaluated. One of the main limitations of viral delivery is packaging size, with the capacity of lentiviruses exceeding AAVs. This makes it difficult to pack large gene sequences or even multiple sequences in one AAV vector, although strategies such as split dCas9 activators resolve some of these issues (Chew et al., 2016). Of note, a split dCas9 strategy was used in a gene therapy for efficient long-term transactivation of cone photoreceptor genes in dysfunctional rod photoreceptors in a mouse model of photoreceptor degeneration. Rods and cones express different genes for their phototransduction cascades. Here, the dysfunctional rhodopsin was replaced with cone opsin, which led to improved retinal function and attenuated retinal degeneration (Böhm et al., 2020). It was pointed out that improvements in the AAV delivery method and especially its efficiency could lead to even higher gain of retinal function. Looking into the potential for clinical use of this method, gliosis or adverse immune responses were not observed as a result of treatment.

In this context, the CRISPRa method has an advantage over other overexpression strategies or gene modification *in vivo*, since the amount of material that needs to be packed is more or less constant. This reduces the effect of gene-size-dependence on viral titers. Targeting of genes is realized by modifying specific gRNAs only. Advances in the design of novel CRISPRa systems allowed for a compact format that makes it possible to use AAVs for targeted delivery of the system in the brain. For example, more compact alternatives to dCas9 derived from *Streptococcus pyogenes*, such as the variant from *Staphylococcus aureus* also allows for the construction of functional CRISPR activators (Lau et al., 2019). This facilitates to design systems based on only a single AAV containing all necessary tools, simplifying production, delivery and efficient activation. Targeting the central nervous system (CNS) by means of systemic delivery requires the use of vectors efficiently crossing the blood-brain barrier. To achieve this, AAV capsids can be engineered to more reliably transduce cells in the CNS (Lau et al., 2019). This study also shows that higher levels of activation can be achieved by targeting genes at different sites using multiple gRNAs. Another tool for delivery is lentiviruses offering a larger loading capacity than AAVs enabling to target a whole matrix of genes simultaneously. Employing this CRISPRa method, it is possible to replicate a gene expression profile observed in an animal model of drug addiction (Savell et al., 2020). Following activation of 16 genes that are most strongly altered by dopamine receptor activation after cocaine administration, using a multiplexed CRISPRa *in vivo* approach, elevated levels of these target genes were observed. This also led to heightened locomotion sensitization in the treated rats. The authors point out that this strategy could be applied to investigate the role of gene expression programs in other settings such as memory formation and neuropsychiatric disorders.

Another study compared the use of lentiviral and AAV systems for *in vivo* overexpression of the Cnr1 gene (Di Maria et al., 2020). Both approaches were successful *in vitro* while *in vivo* only the dual-AAV approach could elicit a measurable response, due to their smaller particle size and resulting ability to penetrate deeper into larger volumes of brain tissue. The results highlight the potential of CRISPRa for the *in vivo* study of synaptic transmission and associated neurological disorders.

## Critical Parameters for Neuronal Differentiation Using CRISPRa and ORF Approaches

**FIGURE 3:**
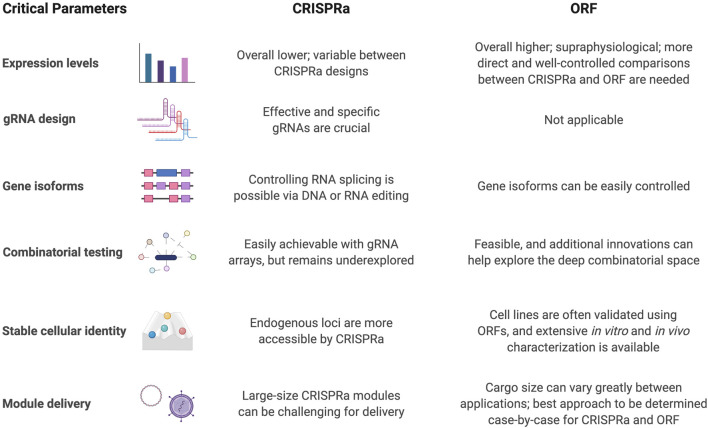
Critical parameters to consider for CRISPRa and ORF-based differentiation strategies.

CRISPRa, as a relatively new approach for upregulation of gene expression, offers unique and complementary features when compared with ORF expression. A set of critical parameters should be considered when comparing CRISPRa and ORF for *in vitro* and *in vivo* approaches (Figure 3). The most apparent aspect to investigate for any gene activation system is how strongly the target genes are induced. A variety of gene activators have been explored with dCas9. For example, dCas9-VPR, dCas9-SunTag-VP64 and VP64-dCas9-VP64 may have a stronger ability than dCas9-VP64 alone to activate gene expression (Tanenbaum et al., 2014; Chavez et al., 2015; Black et al., 2016). In another direct comparison of CRISPRa systems for ASCL1 or NEUROG2 activation, it was reported that SAM outperformed VPR, SunTag, and VP160 (Yang et al., 2019). But a direct and well-controlled comparison between CRISPRa and ORF expression levels is still lacking, and more systematic studies at a genome scale will be needed to compare these systems generally.

Another crucial step for CRISPRa is designing effective and specific gRNAs. Generally, gRNAs are designed to target 50–500 bp upstream of transcription start sites (TSS) (Mohr et al., 2016; Wilson et al., 2018; Liu et al., 2020). It is common, or even preferred, to use multiple gRNAs to activate the targeted locus, especially in pooled screens for many loci (Liu et al., 2018; Black et al., 2020) although this should be weight against increasing gRNA library size, which could impact the signal-to-noise ratio of the screen. The inherent different efficiencies among gRNAs could also provide an interesting source of variation for achieving different levels of gene activation. However, tools for predicting gRNA activity for CRISPRa or general CRISPR applications are still lacking (Wang et al., 2019).

Gene isoforms generated from alternative splicing events and/or TSSs are prevalent in the human transcriptome (Wang et al., 2008), and play important roles in organ development and establishment of cellular identity (Lopez, 1998; Baralle and Giudice, 2017; Imbriano and Molinari, 2018). These can produce transcripts with different protein coding DNA sequences (CDSs) or untranslated regulatory regions (UTRs). By designing gRNAs targeting alternative TSSs, CRISPRa could partially achieve expression of specific gene isoforms, while having limited control in alternative CDSs or UTRs. Several studies have demonstrated the overall technical feasibility of modulating RNA splicing using CRISPR-Cas systems - albeit not directly using CRISPRa - through genome cutting (Mou et al., 2017), base editing (Gapinske et al., 2018), or RNA targeting splicing factors (Du et al., 2020). However, extensive improvements and case-by-case fine tuning is still required. Here, using ORFs coding for TFs with isoform specific CDSs can be an advantage. Previous work comparing the use of four isoforms of ETV2 for endothelial cells differentiation from hiPSCs demonstrated that isoform 2, which has an internal 28 amino acid deletion compared to isoform 1, resulted in doubling of differentiation efficiency to nearly 100% differentiation (Ng et al., 2021), which would be challenging to achieve using CRISPRa systems.

Gene-gene interactions are also important for cell differentiation and function. Because of the short gRNA length, CRISPR systems could be more amenable to combinatorial screening. This is enabled by several factors: the ability to synthesize specific combinations of gRNAs allows for a reduction of the vast combinatorial search space, and the capacity to readily package more gRNAs in tandem into viruses or other vectors compared to ORFs. Despite this notion, combinatorial genetic screening using CRISPRa has yet to be demonstrated convincingly, as only individual genes and select pairs of genes have been screened using CRISPRa. Deep combinatorial screens of many combinations of genes remain unexplored using CRISPRa. One limitation arises from the need for more orthogonal promoters to express gRNAs in order to avoid recombination of the same promoter. It was reported that lentiviral gRNA expression vectors with identical U6 promoters were prone to recombination and loss of gRNAs (Vidigal and Ventura, 2015). Thus, promoters with different sequences, such as human and murine U6 promoters, were needed to avoid this phenomenon. More distinct promoters would be required for even higher multiplexed experiments. Only pairs of gRNAs driven by two promoters have been tested thus far for probing gene interactions in the context of neuronal differentiation (Liu et al., 2018; Black et al., 2020). Work on a similar scale using hundreds of gene pairs has already been performed with paired ORF expression (Tsunemoto et al., 2018). Overall, ORF and CRISPRa-based screens have successfully discovered interactions between TFs for cell programming, and further elucidation of gRNA roles to enhance transcriptional activation and additional promoters for gRNAs would improve the ability for CRISPRa to perform deeper combinatorial screens. In addition to pairwise screening, a three-way combinatorial CRISPR knockout screen has been performed for finding synergistic interactions between druggable targets (Zhou et al., 2020). Cas12a, which can process multiple gRNAs from a single transcript, has been explored and optimized for combinatorial genetic screens (DeWeirdt et al., 2021) in order to avoid the need for multi-step cloning, the problem of barcode recombination, and to harness the potential of large-scale DNA synthesis. Multiplexed gene activation with Cas12a-VPR has also been explored, with room for improvement in terms of activation strength (Breinig et al., 2019; Magnusson et al., 2021). Although CRISPRa may enable more targeted combinatorial screening, it appears that supraphysiological activation using ORFs may be needed until these new developments are adopted for combinatorial CRISPRa screens.

One hallmark of successful cell reprogramming is a stable cell identity *via* the activation of gene regulatory networks. This is important so that reprogrammed cells can maintain their identity without the need for continuous differentiation signals and is one of the marks of truly programmed cell identity. Theoretically, CRISPRa systems are better than ORFs at activating endogenous loci, because gRNAs are targeting the genome directly, while ORFs would need a positive feedback loop to induce expression of the endogenous loci. However, this is only partially supported by a transient transfection experiment that observed higher levels of endogenous transcripts and chromatin modifications in CRISPRa transfected cells than the ORF groups (Black et al., 2016). The differences were not as dramatic when both CRISPRa modules and ORFs were introduced through stable integration (Black et al., 2016). In fact, stable cell identity does not appear to require activation of the endogenous loci of the TF used for programming. For example, ETV2 mRNA was transfected into hiPSCs with all endogenous ETV2 loci knocked out. In these cells, transient ETV2 expression successfully induced stable programming into vascular endothelial cells that were convincingly shown to be functional both *in vitro* and *in vivo* without reapplication of exogenous ETV2 (Wang K. et al., 2020). Furthermore, cells expressing exogenous copies of neurogenic TFs were also able to demonstrate expression of endogenous counterparts and changes in epigenetic modifications around the locus, even without direct targeting. This phenomenon suggests the existence of feedback loops for TF regulation, and that forced expression of key TFs do have the ability to establish a stable gene regulatory network. In practice, it will be important to analyze if the failure to differentiate certain cell types is due to the inability to activate endogenous genetic programs and therefore embark on a better strategy to overcome that hurdle. Recent advances using CRISPR for epigenetic editing (Martella and Fisher, 2021; Nuñez et al., 2021) may further improve endogenous programming. Epigenome editing with dCas9-fused epigenetic modifiers has been used to study how histone acetylation modulates transcription of activity-inducible genes, like Fos and Npas4 (Chen et al., 2019). Manipulating gene expression through epigenetic targeting is a closer mimic to endogenous regulatory processes than targeting gene promoters directly, and may provide more physiological-relevant responses for certain biological questions (Carullo et al., 2020; Yim et al., 2020). Transcripts expressed from the endogenous loci are also subjected to post-transcriptional modifications, which could regulate transcript half-life and spatial localization (Houseley and Tollervey, 2009; Di Liegro et al., 2014).

To activate a set of genes, CRISPRa systems require simultaneous expression of CRISPRa proteins (like dCas9-VP64 or dCas9-VPR) and gRNA cassettes, while ORF strategies need co-delivery of multiple ORFs. The best approach to achieve specific goals should be evaluated on a case-by-case basis. For *in vitro* experiments, if the goal is to achieve strong expression of genes for directed differentiation, direct ORF overexpression is preferred. A combination of multiple promoters and 2A or internal ribosome entry site sequences can enable polycistronic expression of multiple ORFs in the same cell, exemplified by four ORFs for direct cardiac reprogramming (Liu et al., 2017) and nine ORFs for pig germline engineering (Yue et al., 2021). For a large-scale multiplexed screen, CRISPRa systems could be more feasible due to the ease of design and synthesis of gRNA libraries. However, with the newly reported human TFome library (Ng et al., 2021), pooled ORF screens for cell fate engineering are also made more accessible. *In vivo* neuronal programming with TFs is not only important for understanding cellular plasticity and activity, but also an attractive strategy for regenerative medicine. Major roadblocks surrounding *in vivo* CRISPRa applications include delivery of components, strength of activators (Pandelakis et al., 2020) and immunogenicity of CRISPR proteins. Because of the relatively large size of CRISPRa protein modules (4 kb for dCas9 not including activators), optimization of *in vivo* delivery of CRISPRa modules is an active area of research.

## Conclusions and Outlooks

Here, we summarized recent developments using CRISPRa for *in vitro* and *in vivo* neuronal programming. We also highlighted multiplexed neuronal differentiation screens using CRISPRa or ORF expression. Critical technical parameters of these strategies to consider are gene expression levels, gRNA specificity and strength, gene isoform control, scalability for combinatorial testing, stable cellular programming, as well as gene and gRNA delivery. To harness the full potential of CRISPRa for combinatorial screening approaches, careful and rational design of gRNA libraries, scalable readout of perturbation/effects, and statistically robust analysis pipelines are needed. As demonstrated in aforementioned studies, there is a tendency to validate gRNA hits with ORF overexpression as a gold standard in part because of potential off-target effects from gRNA/CRISPR. There are many examples showing that ORF strategies are better suited for generating stable inducible cell lines required for downstream characterizations and applications of the programmed neurons.

As a readout, single cell RNA-seq allows cell type heterogeneity of multiple organs to be resolved at an unprecedented level (Regev et al., 2017; Schaum et al., 2018). Single cell reference atlases of human brain cell types represent an outstanding resource for *in vitro* cell fate studies (Hodge et al., 2019). Due to the advent of tools employing various computational techniques for comparing single cell datasets (Abdelaal et al., 2019; Tran et al., 2020; Pasquini et al., 2021), researchers can now map the identity of novel induced-cells to a much more detailed reference. Yet, neurons can differ by neurotransmitter identity, function, position, physiology, morphology, and molecular profiles. In order to create an adequate neuronal taxonomy, it is required to link relevant phenotype information about neurons with the respective transcriptome profile (Winnubst et al., 2020), as successfully shown for classifying mouse retinal bipolar cells coupling transcriptome and morphology (Shekhar et al., 2016). In line with this, using the spatial and transcriptomic information from the whole human brain (Wang Q. et al., 2020), it is possible to classify cells of *in vitro* derived brain organoids as part of different brain areas, only by measuring the gene expression profile (Fleck et al., 2021). In forward programming screens, the expression of pan-neuronal markers is well suited for systematically testing arising neuronal phenotypes, but each selected TF or combination is likely directing towards different neuronal subtypes and their features need to be further investigated. With the help of high-throughput cellular phenotyping approaches like single-cell and spatial -omics technologies, we are optimistic that ongoing unbiased combinatorial CRISPRa or ORF screens will lead to the discovery of many neuronal differentiation protocols, which will consist of not only novel neurogenic TFs but also new combinations of known neurogenic TFs. As a whole, cellular programming using CRISPRa and ORF technologies will likely enable the production of many neuronal cell types for a plethora of applications.
